# Willoughby Medical School

**Published:** 1835

**Authors:** 


### V. Willoughby Medical School.

A new Medical institution has been lately projected at Chagrin, on Lake Erie, in this state, under the auspices of Willoughby University, of which the Rev. E. F. Willey is President. Lectures were delivered last winter to a class of 25 or 30 pupils; and, as we learn from the public journals of that part of the state, the degree of Doctor of Medicine was conferred on six young gentlemen.

We have also learned, through several different channels, that the Trustees of the Western Reserve College, at Hudson, in the same region, have resolved to institute a Medical Department. Thus the low condition of the Medical College of Ohio, produced by "the Cholera," has driven the people into the necessity of attempting to establish other and better schools.

We do not doubt that, on the shores of Lake Erie, in the eastern part of this state, there will, ere long, be a respectable school; but we should suppose, that Cleveland, from its population and the facility of intercourse between it and Michigan, Upper Canada, the N. W. parts of Pennsylvania, and the S. W. of New York, would be a more elegible point, than either of those selected; and we would respectfully suggest to to the profession on the Western Reserve, to exert themselves in effecting a concentration upon a city—which is evidently destined to become one of the most important in the West.

